# Synergies in exosomes and autophagy pathways for cellular homeostasis and metastasis of tumor cells

**DOI:** 10.1186/s13578-020-00426-y

**Published:** 2020-05-13

**Authors:** Leila Salimi, Ali Akbari, Nassrollah Jabbari, Behnam Mojarad, Ali Vahhabi, Sławomir Szafert, Sadegh Asghari Kalashani, Hamid Soraya, Muhammad Nawaz, Jafar Rezaie

**Affiliations:** 1 Solid Tumor Research Center, Research Institute on Cellular and Molecular Medicine, Urmia University of Medical Sciences, Shafa St, Ershad Blvd, P.O. BoX: 1138, 57147 Urmia, Iran; 2 Department of Biology, Urmia University, Urmia, Iran; 3 Department of Immunology and Genetics, Faculty of Medicine, Urmia University of Medical Sciences, Urmia, Iran; 4 Faculty of Chemistry, University of Wrocław, Joliot Curie 14, 50383 Wrocław, Poland; 5 Department of Medical Laboratory Sciences, Imam Khomeini hospital, Urmia University of Medical Sciences, Urmia, Iran; 6 Department of Pharmacology, Faculty of Pharmacy, Urmia University of Medical Sciences, Urmia, Iran; 7 Department of Rheumatology and Inflammation Research, Institute of Medicine, Sahlgrenska Academy, University of Gothenburg, Box 480, 41346 Gothenburg, Sweden

**Keywords:** Extracellular vesicles, Autophagy, Endosomes, Autophagosomes, Autophagy-mediated exosomes, Autophagy associated tumorigenesis, Cancer cell metastasis

## Abstract

**Background:**

Eukaryotic cells demonstrate two tightly linked vesicular transport systems, comprising intracellular vesicle transport and extracellular vesicle transport system. Intracellular transport vesicles can translocate biomolecules between compartments inside the cell, for example, proteins from the rough endoplasmic reticulum to the Golgi apparatus. Whereas, the secreted vesicles so-called extracellular vesicles facilitate the transport of biomolecules, for example, nucleic acids, proteins and lipids between cells. Vesicles can be formed during the process of endocytosis or/and autophagy and not only act as mediators of intra- and inter-cellular communication but also represent pathological conditions of cells or tissues.

**Methods:**

In this review, we searched articles in PubMed, published between 2000 and 2020, with following terms: autophagy, autophagocytosis, transport vesicles, lysosomes, endosomes, exocytosis, exosomes, alone or in different combinations. The biological functions that were selected based on relevancy to our topic include cellular homeostasis and tumorigenesis.

**Results:**

The searched literature shows that there is a high degree of synergies between exosome biogenesis and autophagy, which encompass endocytosis and endosomes, lysosomes, exocytosis and exosomes, autophagocytosis, autophagosomes and amphisomes. These transport systems not only maintain cellular homeostasis but also operate synergically against fluctuations in the external and internal environment such as during tumorigenesis and metastasis. Additionally, exosomal and autophagic proteins may serve as cancer diagnosis approaches.

**Conclusion:**

Exosomal and autophagy pathways play pivotal roles in homeostasis and metastasis of tumor cells. Understanding the crosstalk between endomembrane organelles and vesicular trafficking may expand our insight into cooperative functions of exosomal and autophagy pathways during disease progression and may help to develop effective therapies against lysosomal diseases including cancers and beyond.

## Background

In eukaryote cells, the intracellular vesicular system plays pivotal roles in the maintenance of cell homeostasis [1, 2], which involves cytoplasmic trafficking of biomolecules inside cells. Different endomembrane organelles such as Golgi apparatus, endoplasmic reticulum (ER), endosomes and lysosomes, in association with cytoskeleton elements are involved in the intracellular vesicular system [1, 3], whereby several molecules participate to maintain homeostasis through the intracellular vesicular system and regulate cells' responses against the internal and external environment. Autophagy is the intracellular vesicular-related process that regulates the cell environment against pathological conditions [4, 5]. Internal (intracellular) vesicles or secreted vesicles can be formed naturally as well as under pathological states during the process of endocytosis or/and autophagy. Importantly, the autophagy and lysosomal/exosomal secretory pathways have been shown to serve as a canal to degrade and expel damaged molecules out of the cytoplasm in order to maintain homeostasis and to protect cells against stress conditions [6]. Autophagy, as intracellular waste elimination system, is a synchronized process that actively participates in cellular homeostasis through clearance and recycling of damaged proteins and organelles from the cytoplasm to autophagosomes and then to lysosomes [7]. The vesicles generated from autophagy are known as autophagosomes and transport the damaged materials to the lysosomes for degradation. Similarly, the vesicles generated from endocytosis and endosomal compartments may either transport the damaged molecules to the lysosomes or expel them out of the cell via exocytosis — so-called exosomes.

Autophagy progressively arises against stress conditions such as hypoxia, nutrient deprivation, organelle damage, and impaired protein [8–10], and plays the central role in adaption to nutrient deprivation, cell death, growth, and tumor progression or suppression. However, at the basal level, autophagy contributes to control biological process, quality of proteins and organelles, and eventually provides a safe environment for cells [11]. This process is capable of suppressing tumorigenesis through preventing tumor cells proliferation and inducing apoptosis, however, there is also evidence that it facilitates the tumorigenesis by supporting tumor cells proliferation and metastasis [12, 13]. Studies have indicated that common proteins participate to mediate the crosstalk between exosomes biogenesis and autophagy. This crosstalk controls tumor cell function and fate. Under physiological and pathological conditions, the coordination between exosome–autophagy networks serves as a tool to conserve cellular homeostasis via the lysosomal degradative pathway and/or secretion of cargo into the extracellular milieu [14, 15]. In this review, we describe the biogenesis of exosomes in linkage with autophagy, placing a particular focus on shared roles of exosomes and autophagy that are pivotal in cancer biology. Additionally, we discuss the clinical applications of exosomes and autophagy in cancer diagnosis.

### Characteristics of autophagy and autophagic biological process

Autophagy is defined as a regulated process inside almost every cell type activated against various stress conditions such as starvation, hypoxia, oxidative stress, protein aggregation, and endoplasmic reticulum stress [16, 17]. Additionally, autophagy regulates energy balance in the biological system and plays a central role in regulating cell survival and differentiation [7]. The autophagy is a way to eliminate impaired and misfolded proteins, protein aggregates, damaged organelles, and intracellular pathogens, which are encapsulated into autophagosomes and finally fuse with lysosomes for subsequent degradation [18].

**Fig. 1:**
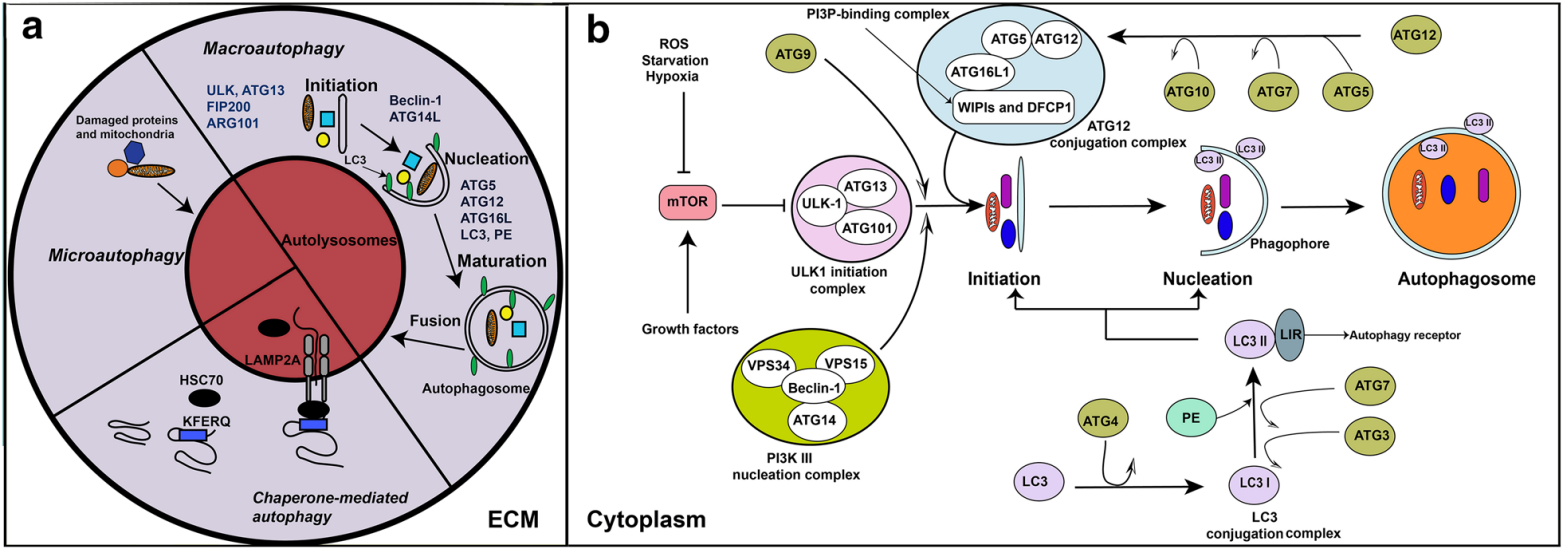
A schematic illustration of three types of autophagy and key regulatory molecules of autophagy flux inside cell. **a**: Three types of autophagy may occur in cell; microautophagy, chaperone-mediated autophagy, and macroautophagy [19, 20]. Microautophagy is the process during which damaged biomolecules are directly sorted into lysosomes. In chaperone-mediated autophagy, HSC70 identifies proteins containing specific motifs (KFERQ) and sorts them into lysosome through interaction with LAMP2A molecules placed on lysosome membrane. Macroautophagy mediates the lysosomal degradation of damaged proteins and organelles through 4 steps including initiation, nucleation, maturation, and finally fusion the autophagosome with lysosomes. Several proteins such as ULK, ATG13, FIP200, ARG101, Beclin-1, ATG14L, ATG5, ATG12, ATG16L, LC3, and PE, in different steps, mediate the formation of autophagosome [19, 20]. **b**: Once autophagy is induced, cytoplasmic dysfunctional molecules are encapsulated via double membranes, beginning from the formation of the phagophore to the autophagosomes, which consequently fuse with lysosomes and then their cargo is degraded [24]. Several ATG-associated assemblies including ULK-1 initiation complex, the PI3K III nucleation complex, the ATG12 conjugated complex, and the LC3 conjugation complex are involved in autophagy flux, which finally direct cytoplasmic dysfunctional molecules into lysosomes [24]. Stress condition such as starvation, energy depletion, reactive oxygen species (ROS), and hypoxia inhibit mTOR and growth factors act as activators of mTOR. Inhibition of mTOR activates the ULK-1 initiation complex which, in turn, mediates initiation of autophagy flux. In this scenario, AGT9 and the PI3K III nucleation complex collaborate with the ULK-1 initiation complex and progress initiation step of autophagy [24]. These complexes are supported by the ATG12 conjugation complex and the LC3 conjugation complex for completing initiation step and formation of phagophore in nucleation step. In order to formation of the ATG12 conjugation complex, ATG12 attaches to ATG5 and ATG16L1, and then the PI3P-binding complex (WIPIs and DFCP1) joins them to form the ATG12 conjugation complex. Formation of the ATG12 conjugation complex then facilitates connection of LC3 conjugation complex to newly formed phagophore in nucleation step, at this moment, ATG4 catalyzes the formation of LC3-I from LC3. Next, conjugation of PE with LC3-I, in presence of ATG7 and ATG3, forms LC3-II. This molecule is assimilated into phagophore and autophagosomal membranes, where LC3-II interacts with cargo receptors, which harbor LIRs [24]. DFCP1, zinc-finger; ECM, extracellular matrix; FYVE domain-containing protein 1; LC3, microtubule-associated protein light chain 3; LIRs, LC3-interacting motifs; PE: phosphatidylethanolamine ULK-1, Unc-51-like kinase 1; WIPIs, WD repeat domain phosphoinositide-interacting proteins

At the mechanistic level, the autophagy is considered a multi-step process that occurs by initiation, membrane nucleation, maturation and finally the fusion of autophagosomes with the lysosomes. The autophagy can be categorized into three major types such as (I) microautophagy, (II) macroautophagy, and (III) chaperone-mediated autophagy (CMA), occurring during the degradation and recycling of dysfunctional proteins and organelles [19, 20] (Fig. 1a). The autophagy flux/turnover is mediated by different stimuli. For example, hypoxia, reactive oxygen species (ROS), and starvation could contribute to induce autophagy [21]. The nutrient availability has been shown to play a key role in the autophagy process through the mechanistic targeting of rapamycin (mTOR) signaling pathway. In cells which are rich in nutrients and growth factors, the mTOR complex 1 (mTORC1) inhibits the autophagy through phosphorylation and inhibition of the autophagy-initiating kinase Unc-51-like kinase 1(ULK1) (Fig. 1b). Contrarily, the nutrient starvation inhibits the mTORC1, which in turn induces autophagy [22].

Under normal conditions, the mTOR blocks autophagy through phosphorylation of Atg13. In turn, this process inhibits the interaction of Atg13-ULK1-Atg17 complex. In addition, mTOR phosphorylates ULK1 and blocks the interaction of ULK1 and AMPK [23]. Conversely, under stress conditions such as starvation, hypoxia, and ROS production, where mTORC1 is inhibited, the formation of the ULK1-Atg13-Atg17 complex induces autophagosome formation [24]. As such, at the membrane nucleation step, the Atg9 and PI3k III nucleation complex (Beclin-1, Atg14, VPS34, and VPS15) coordinates with ULK1-Atg13-Atg17 complex, which consequently induces membrane nucleation and phagophore formation (pre-autophagosome compartment) [25]. At the same time, using a NEM-Sensitive Factor (NSF) Attachment Protein Receptor (SNARE) protein, syntaxin 17 molecules and Atg14 are settled on the mitochondria-ER connection border [26]. Consequently, different types of ATG proteins contribute to form phagophore. For instance, Atg5, Atg7, and Atg10 support Atg12 to conjugate with a multi-molecular complex (Atg12-Atg5-Atg16L-WIPIs-DFCP1) known as Agt12 conjugation complex, which participates in the formation of the phagophore membrane [27].

In the initiation phase, LC3-Agt13 conjugation complex contributes to ULK1 activation and consequently induces autophagosome biogenesis. However, during the maturation step, LC3-ATG8 mediates closure, fusion, or transport of the autophagosomes [28]. As shown in Fig. 1b, in LC3 conjugation complex, initially Atg4 and Atg3 molecules catalyze LC3β-I generation and then phosphatidylethanolamine (PE) catalyzes the formation of LC3β-II from LC3, which is maintained in associated with phosphatidylethanolamine. LC3β-II binds to autophagy receptors and phosphatidylethanolamine along with Atg9 and ULK complex on both sides of the membrane [18]. Once autophagosome is formed, LC3β-II disassociates from the external surface of the membrane. In the final step, autophagosomes containing different cargo fuse with the lysosomes and form the autophagosomes–lysosome hybrid vesicles, which now called autolysosomes. The fusion with lysosomes is dependent on the participation of different molecules such as microtubules, Rab7, LAMP1/2, SNAREs, and ESCRT [29, 30]. Hydrolysis enzymes located in lysosomes could degrade the cargo on arrival [17, 18].

Growing evidence suggests that proteins and organelles as well as p62, a ubiquitin-binding protein associated with autophagosome is degraded, whereas LC3 could be recycled to another autophagy event [9, 31]. LC3β has been considered as an autophagy flux marker due to association with the autophagosome. However, LC3β is also present on macropinosomes as single-membrane phagosomes or in a cellular mechanism called LC3β-associated phagocytosis (LAP). At this point, the Agt12 conjugation complex plays a pivotal role in directing LC3β to the phagosome membrane [32]. LAP could have a role in facilitating the fusion of phagosome with lysosomes, and thus may accelerate the degradation rate of unwanted molecules [33]. However, a recent report indicates that lysosomal inhibition has no effect on the LC3β lipidation at single-membrane endosomes which may imply the non-degradation role of a LAP-like compartments [34].

Eukaryotic cells contain a complex intracellular organization that distinguish them from prokaryotic cells. In these cells, specific cellular functions are classified into the nucleus and other organelles enclosed by intracellular membranes [1, 35]. Intracellular vesicular system contributes to the intra- and trans-organelle communication. This system is complex and consists of formation, fusion, division and trafficking of membranous vesicles, which is essential for regulating the basic and specialized functions in cells [1, 35]. The intracellular vesicular system regulates cellular uptake/internalization and organization of foreign pathogens and substances for degradation and that of nutrients to metabolic processing. Newly produced molecules and complexes are localized to subcellular locations such as endosomes and lysosomes, where degradation of toxic molecules occurs. Additionally, the process encompasses the intra- and trans-organelle messages, signaling cascades, modification and recycling of biomolecules such as proteins and lipids [1, 35]. Vesicular processes are dynamic and interconnected within subcellular compartments. Among them, the well-known vesicular processes are autophagy-related vesicles and endosome-derived vesicles i.e. exosomes [16].

### Exosome biogenesis and autophagy: synergies in degradation, recycling and secretion

Almost every cell type secretes nano to micro-sized vesicles into the extracellular environment and are collectively termed as extracellular vesicles (EVs) [36]. Well-described class of EVs is the exosomes, which originate from endosomal compartments and share several lines of linkages with endocytosis, lysosomal degradation and autophagocytosis (discussed in later sections). In the endosomal pathway, the cargo or biomolecules internalized through the plasma membrane are engulfed in early endosomes which are either recycled to the plasma membrane or localized to lysosomes or sorted into late endosomes also known as multivesicular bodies (MVBs). In the latter case, the cargo that is not directed to lysosomes for degradation is sorted into intraluminal vesicles (ILVs) of MVBs for subsequent secretion into extracellular milieu via exocytosis when MVBs fuse with the plasma membrane [37, 38].

**Fig. 2:**
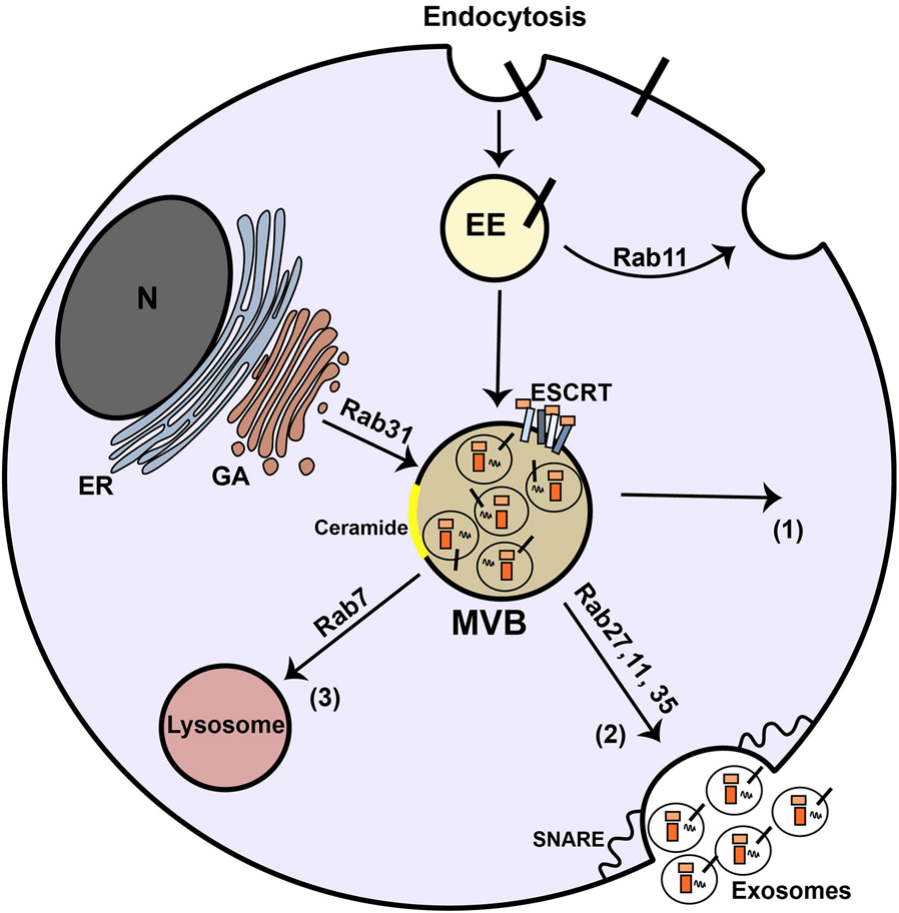
Formation of exosomes inside cell. Exosomes are nano-sized vesicles generated from endocytic pathway [5]. They are formed from inward budding of the membrane of multivesicular bodies (MVBs), late endosomes of endocytic pathway, through ESCRT-dependent machinery which involves assortment of ubiquitinated cargo. In addition, different proteins and lipids including CD63 and ceramides mediate exosome biogenesis which known as ESCRT-independent machinery [5, 38]. MVB’s cargo is provided with different sorting molecules located on MVB’s membrane, cytoplasm, and Golgi apparatus. Different Rab-GTPases such as Rab7, Rab11, Rab27, and Rap35 preferentially mediate intracellular trafficking of MVBs. MBVs may back fuse to the plasma membrane and recycle biomolecules to the plasma membrane or present specific biomolecules (such as major histocompatibility complex (MHC) proteins) (1). SNARE and Rab-GTPase (Rab11, Rab27, and Rab35) proteins facilitate the fusion of MVBs with the plasma membrane in order to release exosomes into extracellular environment (2). In degradation pathway, MVBs can fuse with lysosomes for hydrolyzing their cargo (3). EE, early endosome; ER, endoplasmic reticulum; GA, Golgi apparatus; N, nucleus

Exosome biogenesis is a tightly regulated process, which involves endosomal sorting complexes required for transport (ESCRT)-dependent machinery and ESCRT-independent machinery (Fig. 2a). The ESCRT machinery consists of four complexes (ESCRT 0, ESCRT I, ESCRT II, and ESCRT III) and accessory proteins located on the cytoplasmic side of MVB’s membrane, which contribute to the formation of ILVs inside the MVBs and sort the ubiquitinated proteins cargo into ILVs in an ATP-dependent manner [5, 39]. Some of the ESCRT subunits and accessory proteins such as hepatocyte growth factor-regulated tyrosine kinase substrate (HRS), apoptosis-linked gene 2-interacting protein X (ALIX), and tumor susceptibility gene 101 (TSG101) are released through exocytosis and are commonly considered as exosomal markers [5]. Accumulating evidence indicates that exosomes from ‎different cell origin represent the common markers, for instance, CD63, CD9, ‎CD81, CD82, ALIX, and TSG101 [37, 40, 41].

In contrast, the ESCRT-independent machinery involves molecules other than ESCRT subunits, such as tetraspanins, lipids, and proteins as well as membrane typology (microdomains), which contribute to inward invagination of MVBs, ILV formation and exosome sorting [5] (Fig. 2). Ceramide, a waxy lipid molecule, is a key molecule in generating ILVs from MVBs [42]. Indeed, proteolipid proteins (PLP) are engulfed into ILVs in the absence of ESCRT machinery via lipid raft-based microdomains that are enriched in sphingolipids, from which ceramides are generated in the presence of an enzyme named sphingomyelinase. The ceramide promotes the joining of microdomains and induces ILVs formation [42]. Concurrently, Edgar et al. reported that CD63 plays a pivotal role in the formation of MVBs in different cells including those in HeLa cells [43]. Moreover, in HEK293 cells, the expression of CD9 and CD82 molecules promotes the formation of exosomes containing β-catenin through ceramide-dependent way [44]. Additionally, the key role of phospholipase D2 has also been reported in lipid assisted exosome biogenesis. Indeed, phospholipase D2 generates phosphatidic acid (PA) from phosphatidylcholine, which induces exosome formation similar to ceramide [45]. ESCRT-dependent or independent mechanisms contribute to producing exosomes, however, it remains unclear whether both mechanisms operate in a synergy or separately, and whether heterogeneous subpopulations of &;MVB-derived exosomes and their composition are the result of different machineries in these pathways [5, 46].

Additionally, the exosomes cargo may also consist of molecules sorted from the intracellular vesicular system such as Golgi apparatus/vesicles, endocytosis pathway and/or from autophagosomes [47]. In parallel events, it is thought that the plasma membrane, Golgi apparatus, and endoplasmic reticulum governed by autophagic proteins participate to the initial formation of autophagosome, which engulfs dysfunctional bio-molecules for the downstream process [48].

The MVB trafficking also shows similarities with autophagy for lysosomal degradation, especially both processes share SNARE, Rab7 and ESCRT. Rab-GTPases mediate intracellular trafficking of vesicles, as well as membrane fusion in the endocytic and exocytic pathway [48]. In fact, the mature MVBs have three fates including degradation, back-fusion with the plasma membrane (recycling), and secretion (Fig. 2). In degradation pathway of ILVs, Rab7 regulates MVB trafficking to lysosomes [49], where lysosomal enzymes degrade MVB cargo. The degraded biomolecules are recycled for cellular consumption. In autophagy pathway, Rab7 regulates formation and trafficking of double-membrane vesicles known as autophagosomes, which could fuse with lysosomes for degradation of toxic cargo [18]. Through the degradation vesicles (autophagosomes), particularly under stress conditions, the monomers of carbohydrates, proteins, and lipids comprising sugars, amino acids, and fatty acids respectively are recycled and are consumed for cell maintenance and survival [17]. MVBs may also back-fuse with the plasma membrane by Rab4 and Rab11 and decorate the plasma membrane with surface molecules including the major histocompatibility complex (MHC) and receptors. Alternatively, in the secretory pathway, MVBs fuse with the plasma membrane to release ILVs as exosomes into the extracellular milieu. In this regard, SNAREs in cooperation with trafficking proteins i.e. Rab-GTPases participate in the fusion events of MVBs with the plasma membrane (Fig. 2).

Besides the degradation function, the autophagy machinery contributes to the export of cytosolic proteins and cytokines, which is different from the conventional secretion pathway of the Golgi/ER/plasma membrane axis. Previous studies have confirmed the autophagy-dependent secretion of interleukin 1β (IL-1β) from the cytosol to the extracellular matrix by autography machinery [50, 51]. In summary, in the exosomal pathway, excessive molecules on the plasma membrane and internalized molecules are directed into endosomal vesicles, step by step. This may exhibit the similarity with autophagy steps, for packaging unwanted biomolecules into vesicles [39, 52]. In this regard, the MVBs are responsible for processing the cargo and sorting it either for degradation, recycling or exocytosis.

Based on the available evidence it can be speculated that MVBs are transient structures where cell condition decides their fate for degradation versus secretion. Similarly, the fate of autophagosomes which contain biological cargo may also be affected by cell condition. For instance, in lung epithelial cells, IFN-γ stimulation could lead to the secretion of Annexin A2 (ANXA2), a phospholipid-binding protein, via autophagy pathway [53]. This is very interesting to note that the autophagosomes can shift from conventional degradation pathway to secretory one, and thus may share similarity with exosome-based secretion. It is likely that these processes could have been evolved to perform the same functions in cells but in different forms.

### Crosslink between exosome biogenesis and autophagy pathways

#### Molecular mechanism of exosome biogenesis related to autophagy pathways

**Fig. 3:**
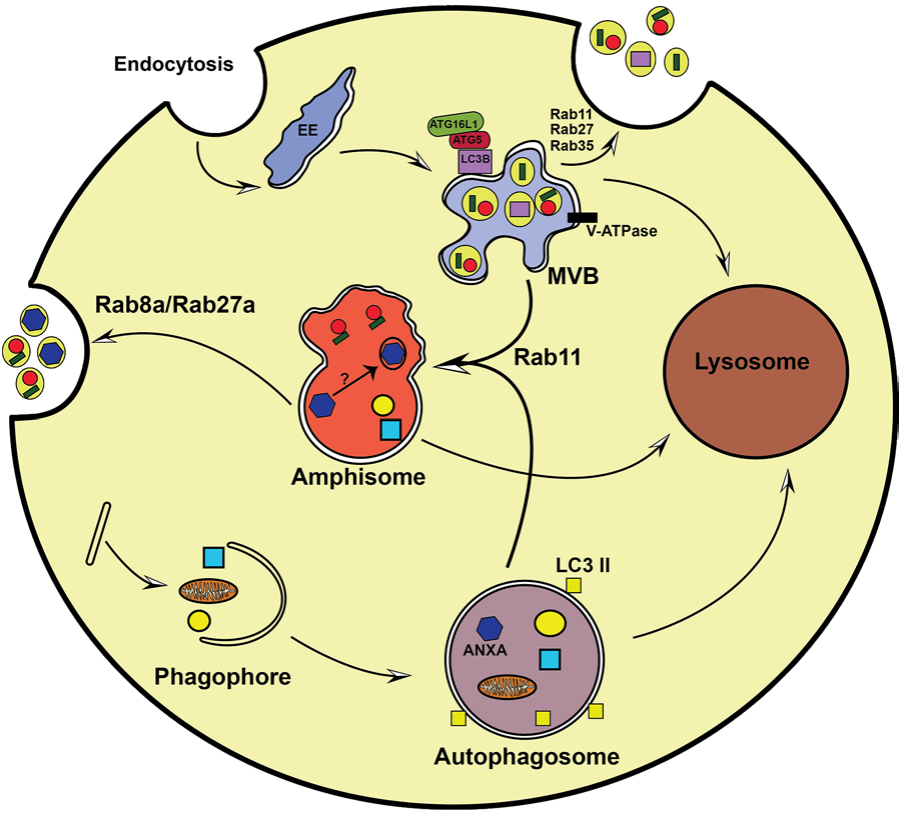
Crosstalk between exosome biogenesis and autophagy. Link between exosome biogenesis and autophagy pathways exists not only at molecular level but also at membranous vesicles such as amphisomes. In this cooperative action, various Rab-GTPase proteins including Rab8a, Rab11, and Rab27 control the movement of vesicles between exosomal secretory pathway and autophagy at the cytoplasm. Autophagic proteins including LC3β, ATG5, and ATG16L1, on the MVB’s membrane, contribute to generate exosomes. Then the autophagic cargo can be secreted into extracellular milieu via exosomes. Additionally, the MVBs may fuse with autophagosome to make hybrid vesicles named amphisomes. Amphisomes cargo may be degraded by lysosomes or alternatively may fuse with the plasma membrane and secrete cargo into extracellular milieu. Amphisomes participate in packaging of annexin A2 (ANXA2) into exosomes; however, which cargo received from autophagosomes may sort into exosomes in amphisomes is still remains a mystery

Different autophagic molecules may lead to exosome biogenesis and secretion (Fig. 3) [54, 55]. For example, C-terminus (GIPC) and G alpha interacting protein (GAIP), the key autophagy regulators, have been shown to increase exosome biogenesis in pancreatic tumor cells [56], indicating the autophagy-mediated exocytosis. The pivotal role of ATG16L1 and ATG5 in exosome biogenesis has been well-established [54]. ATG5 contributes to detachment of V 1/V 0 –ATPase (vacuolar proton pumps) from the MVBs, thereby inhibiting the acidification of MVB-lumen [54]. This process directs the fusion of MVBs with the plasma membrane instead of lysosomes. Indeed, inhibition of ATG16L1 and ATG5 markedly diminishes exosome secretion and lipidated LC3β in exosomes. Likewise, the V-ATPase or lysosomal inhibitors prevent exosome secretion, indicating the crucial role of pH within the MVB lumen for determining the fate of exosomes. Although, the exact role of LC3β in exosome biogenesis is not clear, however, it has been shown that it is located on the inside face of ILVs, suggesting the LAP-like lipidation process at the MVB surrounding membrane or at the inward budding sites of MVB membrane that consequently generates ILVs. Accordingly, secretion of the LC3B-positive exosomes implies that the LAP-like mechanism participates in generating non-degradative ILVs [54]. Given together, ATG16L1 and ATG5 protect MVBs from lysosomal degradation and direct them into the secretory pathway rather than the lysosomal pathway.

Interestingly, the ATG12–ATG3 complex, which mediates LC3β conjugation, regulates exosome biogenesis through interaction with ALIX, a protein that cooperates with ESCRT-III complex [55]. Of note, ALIX can directly interact with exosomal cargo, which offers a sign of discrepancy between the exosomal secretory pathway and the lysosomal degradation pathway [57, 58]. Murrow and colleague showed that inhibition of ATG12–ATG3 complex may change MVB shape and disrupt the late-endosome trafficking and thus reduce the exosome biogenesis. In addition, the authors found that ALIX inhibition also decreases the autophagy flux, indicating a regulatory cross-link between exosome biogenesis and autophagy pathways.

ALIX or the ATG12–ATG3 complex knockdown dose not inhibit starvation-induced autophagy that indicates the involvement of various complexes governing basal and stress-induced autophagy [50]. A work by Bader and co-workers confirmed that ATG9, a transmembrane ATG, contributes to the generation of ILVs in *Drosophila*. In contrast, the inhibition of ATG9 inhibited the autophagy flux and diminished the ILVs content of amphisomes and autolysosomes. However, it remains unknown whether these ILVs are secreted as exosomes [59].

Another complex, which shares a key role in exosome biogenesis and autophagy pathways is the class III PI3K complex. In mammalian cells, this complex is composed of Beclin-1, VPS34, p150, and different accessory proteins and is shared between autophagy and endocytosis processes. PI3K is essential for endocytosis and autophagy through phosphorylation of phosphatidylinositide to generate PI (3)P, which mediates membrane trafficking. In this regard, the presence of ATG14L in PI3K complex regulates autophagosome development, whereas UVRAG engagement mediates endosome maturation, demonstrating the determinative function of this complex [60]. Furthermore, the PI3K complex, containing Run domain Beclin-1-interacting and cysteine-rich domain-containing protein (Rubicon), not only mediates LC3-based phagocytosis but also participates to block the endocytosis and autophagy [61]. Of note, the distribution of PI3K complex through suppressing Beclin1 decreases both autophagy and exosome biogenesis in human chronic myeloid leukemia (CML) cells [62].

### Cytoplasmic membrane system, exosome biogenesis and autophagy

The cytoplasmic membrane system represents the crosslink between exosome biogenesis and autophagy processes largely involving the amphisomes, yet another type of degradative vesicles (Fig. 3). These transient compartments are formed through membranous hybridization of autophagosomes and MVBs, which ultimately combine with lysosomes and undergo degradation (Fig. 3). The formation of amphisome may negatively regulate the coordination between exosome secretion and autophagy. For example, rapamycin or starvation treatment of K562, an erythroleukemic cell line, promotes autophagy and MVB-autophagosome fusion and declines exosome secretion [63], probably supporting cell’s effort to save energy. Interestingly, inhibition of exosomes secretion alternatively results in the trafficking of MVBs towards autophagy pathway. Villarroya-Beltri et al. examined the ubiquitin-like protein ISG15 named as ISGylation in vitro and in mice models. They showed that ISGylation of TSG101, an ESCRT-I supporting protein may cause aggregation and degradation of proteins and thus may decrease MVB and exosome biogenesis [64]. However, inhibition of lysosome-endosome axis through blockage of autophagy (e.g., by abafilomycin A1) may enhance the exosome release. This indicates that autophagy mediates the lysosome-based degradation of MVBs containing ISGylation-induced aggregates [64].

Hurwitz et al. showed that knockout of CD63 resulted in the formation of atypical endocytic vesicles inside cells, which consequently were degraded by autophagic clearance system, however, inhibition of autophagy could moderately elevate the exosome biogenesis [65]. These findings elucidate the key role of autophagic degradation in the regulating exosomal pathway. Besides a role in the degradation pathway (described above), the LC3β colocalizes with the endosomal markers such as Rab11, RAB7, and EEA1, on amphisome-like compartments. As such, it contributes to the generation of ROS, which mediates the secretion of mucin granules in mice intestinal goblet cells [66]. Similarly, in lung epithelial cells, amphisomes contribute to the secretion of exosomes bearing ANXA2 [53].

IFN-γ treatment could also initiate the autophagy and induces the fusion of CD63, LC3β, and ANXA2 with amphisomes. Subsequently, RAB11 and RAB27A control the fusion of amphisomes with the plasma membrane and then secretion of amphisomes cargo into the extracellular space [53]. Notably, this secretory pathway is different from the exosomal secretion. Indeed, autophagy-based secretion of IL-1β is depended on functional MVBs [67], however, autophagosome–lysosome fusion is independent of MVBs [51], indicating that LC3β-positive IL-1β bearing vesicles may fuse directly with the plasma membrane. In this context, RAB8A mediates the IFN-γ-induced exosome biogenesis [5], and autophagy-dependent IL-1β positive vesicle secretion [4].

Exosome secretion pathway and autophagy flux may corporate to protect the cell from stress conditions [68]. However, it appears that these pathways collectively orchestrate the dynamics of intracellular removal processes, where each pathway may occur in alternative forms to complement the insufficiency of the other. As such, the unwanted MVBs with damaged material may be directed to autophagy pathway, and likewise, the defects in autophagy may promote fusion of MVBs with the plasma membrane to release toxic/damaged material via exosomes [69].

### Uptake routes of exosomes and other extracellular vesicles

Once released into extracellular space, exosomes and other EVs are distributed through the bloodstream and other bio-fluids in order to deliver their cargo to neighboring and distantly located cells [70] and induce phenotypic changes in recipient cells. Three pathways for EVs uptake have been proposed by which they can affect the target cell function [71, 72]. These include (a): direct fusion with the cell, (b): receptor/ligand interaction, and (c): internalization pathway. In direct fusion, EV membrane combines with the plasma membrane of target cells similar to the conventional membrane fusion process. Consequently, EV cargo enters directly to the cytoplasm of the target cell. However, during the receptor/ligand interaction (docking), the receptors such as intercellular adhesion molecule 1 (ICAM-1) present at the EV membrane, interact with the receptors located on the target cell membrane. For instance, EV-associated ICAM-1 interacts with lymphocyte function-associated antigen 1 receptor and activates the downstream molecular cascades inside the recipient cell [73]. Alternatively, EVs could be internalized by unspecific macropinocytosis or micropinocytosis and may induce downstream signaling pathways in target cell [74, 75].

In addition to these pathways, it is suggested that EV membrane-related molecules may be activated by enzymes present in the extracellular matrix. Consequently, the activated molecules bind to receptors on target cells as a ligand [76]. Given any means of uptake or engulfing of EVs by recipient cells, the content of EVs can foster the dictated patterns of trans-regulation in recipient cells [59]. This refers to the induction of genetic regulation, cellular reprogramming and genomic instability elicited in recipient cells, which ultimately may lead to pathological conditions including generation of cancer-initiating cell phenotypes, and resistance to chemotherapies [77].

### Coordinated roles of exosomes and autophagy in tumor

#### Exosomes biogenesis and activation of autophagy during tumorigenesis

In tumor cells, both autophagy and exosome secretion are accelerated. Nutrient deprivation and hypoxia (which are present in the tumor environment) induce autophagy flux, which defends against inflammation and necrosis [78, 79]. Additionally, a hypoxic tumor microenvironment induces exosomes biogenesis, which enables tumor cells to survive under stress conditions [80]. For example, in breast cancer cells, the stress conditions such as hypoxia induces the exosome secretion [81] as well as the autophagy flux [82]. It is well established that endoplasmic reticulum stress induces autophagy flux in various types of tumor and non-tumor cells [83]. In this context, endoplasmic reticulum stress has also been shown to increase the number of MVBs in the cytoplasm of Hela cells and to elevate exosome release through IRE1a and PERK signaling pathway [84]. Using in vitro rotenone-induced mitochondrial injury experiment, Kumar and colleagues found that endosomal tetraspanins such as CD63, CD81, and CD9 as well as ATG7 are upregulated in prostate and breast cancer cell lines, which indicate the increased activity of both autophagy and exosome biogenesis in response to cancer-mediated stress condition [68]. Further evidence comes from a knockdown study of Bhattacharya’s team, where the authors confirmed that GAIP-interacting protein C-terminus (GIPC) concurrently controls exosomal and autophagy pathways in pancreatic cancer cells [56]. Of particular note, in prostate cancer cells, the FYVE-type zinc finger-containing phosphoinositide kinase (PIKfyve) contributes to control the fate of proteins, which are sorted into the exosomal secretory pathway or autophagic degradation pathway [85]. PIKfyve may serve as homeostasis molecule and enables cancer cells to survive/adapt under stress conditions. A study by Qi and colleagues confirmed that mesenchymal stem cells (MSCs)-derived exosomes were capable of inducing the Hedgehog signaling pathway in gastric cancer osteosarcoma cell lines, which increased tumor growth rate [86]. Additionally, exosomes obtained from MSCs contain MMP-2 molecules that contribute to the remodeling of the tumor microenvironment and cell growth [87].

Besides exosomes contribution in intercellular communication, the autophagy machinery also participates in interactions among tumor cells and non-tumors cells in a given tumor microenvironment. The autophagy-based release of IL-6 participates in the formation of mammosphere, which is vital in cancer stem cell survival. Exosomes can also influence the dynamic of autophagy in cancer cells. For instance, exosomes from breast cancer cells induce autophagy flux in breast epithelial cells in vitro. In this context, exosomes taken up by breast epithelial cells upregulate the ROS production, which in turn induces autophagy influx and subsequently autophagy-related secretion of pro-tumor growth factors from recipient cells [88]. In the tumor microenvironment, stromal cells release the cytokines and growth factors, which facilitate cancer cell growth through the autophagic secretory pathway [89, 90]. As such, the crosstalk between exosome biogenesis and autophagy orchestrates the intra-tumor communication.

### Exosomes and autophagy in cancer cell invasion

**Fig. 4:**
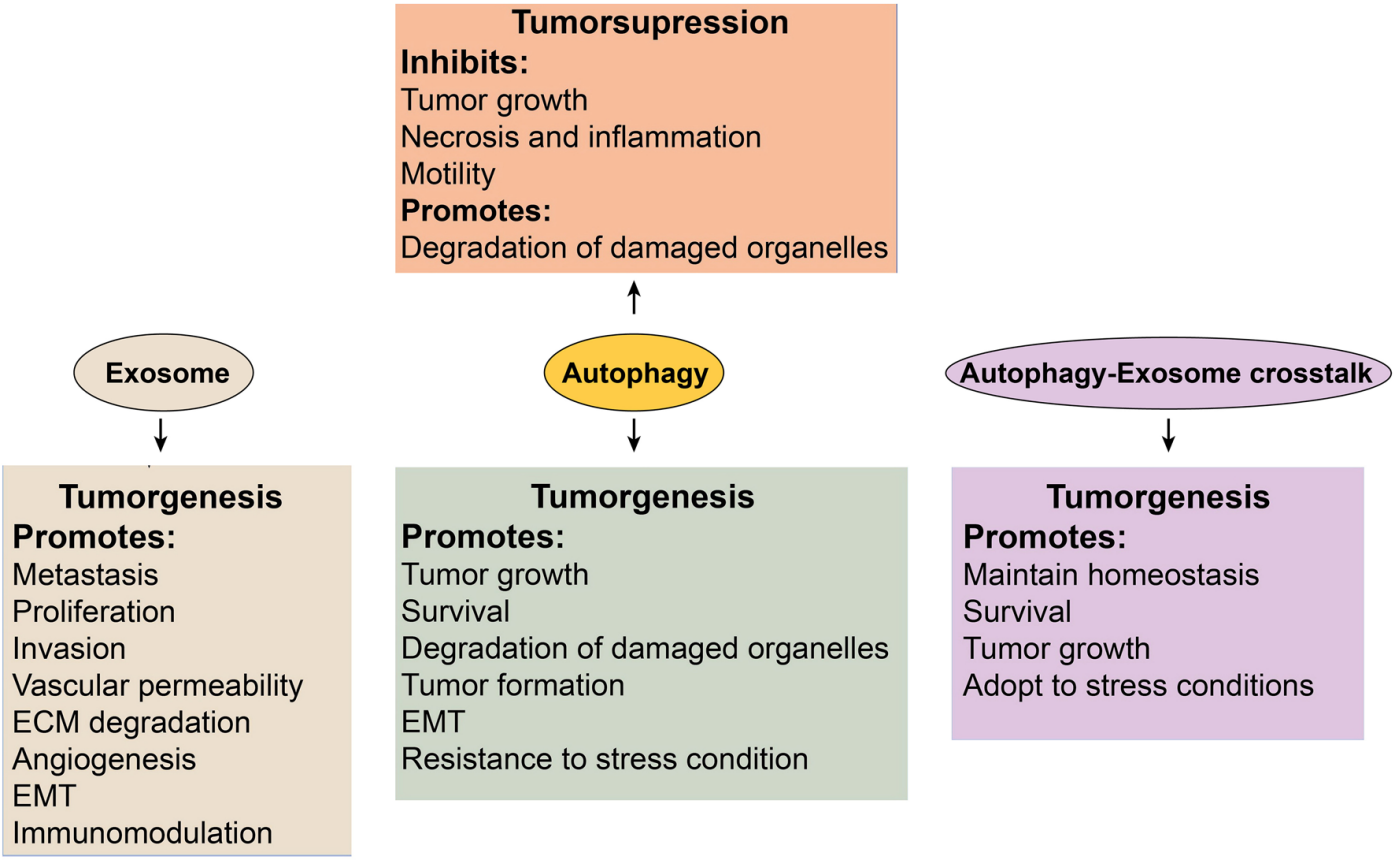
A schematic diagram of key roles of exosomes, autophagy, and autophagy-exosomes crosstalk in cancer metastasis

Metastasis, i.e. migration of cancer cells from the site of origin to secondary tissues, is one of the hallmarks of cancer [91]. Tumor cells actively produce exosomes which not only represent the aggressive feature tumor but also promote different pathological aspects of cancers [92] (Fig. 4). The possibility that invasion can be promoted by tumor-derived exosomes was confirmed by Hood and colleagues, where fluorescent labeled exosomes when co-cultured with endothelial cells, they passively reached to cells and induced the formation of endothelial spheroids and endothelial sprouts, which finally promoted metastasis [93]. Similarly, the oncogenic receptor EGFRvIII transferred by microvesicles from glioblastoma cells enhanced the cell proliferation and tumor invasion [94]. Additionally, exosomes containing matrix metalloproteinase-13 (MMP13) promote nasopharyngeal carcinoma cells metastasis through the degradation of the extracellular matrix (ECM) [95].

However, autophagy is a bilabial process. In normal cells, it contributes to suppressing tumorigenesis, but in transformed cells, it promotes tumorigenesis (Fig. 4). In a progressive metastasis stage, autophagy shows a pro-metastatic property, therefore, promotes cell survival and migration toward secondary tissues [78]. As a result, inhibition of autophagic genes including LC3 and Beclin-1 inhibits the proliferation, movement, invasion, and increases the apoptosis in breast cancer cells [96]. By recruiting oncogenic Ras mutations in cancerous cells, the autophagy favors the tumor progression through protecting mitochondrial integrity [97].

### Exosomes and autophagy in the extracellular matrix remodeling

It is well-known that miRNA cargo of exosomes participates in shaping tumor cell phenotype in recipient cells, and formation of pre-metastatic cells. In this regard, in animal xenograft cancer models, breast carcinoma-derived exosomes enhanced the metastasis potential of indecisively metastatic cells and participated in homing these cells in distantly located tissues. These responses may partially occur due to miR-200 molecules transferred via exosomes [98]. Breast cancer-derived exosomes are enriched with miR-105, which suppresses ZO-1 protein in endothelial cells, and increases the vascular permeability [99]. In addition, exosomes from metastatic rat adenocarcinoma BSp73ASML have been shown to transfer miR-494 and miR-542-3p, which target the cadherin-17 and MMPs expression for seeding the pre-metastatic niche [100].

There is increasing evidence that suggests a pivotal role for prostate cancer exosomes in ECM degradation. These exosomes contain various miRNAs, such as, miR-21-5p, miR-139-5p, and miR-100-5p, which control the expression of a panel of MMPs proteins (e.g. MMP2, MMP9, and MMP13) and promote tumor invasion [101]. Loss of ECM causes cell apoptosis, which called anoikis, confirming that the autophagy reinforces metastatic cancer cells against anoikis [102]. Furthermore, autophagy promotes the detachment of cancer cells from ECM and the suppression of β1 integrin, an adherent molecule [103]. In a mouse lung metastasis model, suppression of autophagy diminished the metastasis indexes, proliferation, and anoikis resistance of hepatocellular carcinoma cells [104].

Autophagy may provide a mechanism that detaches cells from the basal matrix at the same time protecting them from anoikis. It was reported that in the early stage of cutaneous melanoma cancer, down-regulation of ATG5 gene promotes the cancer-cell proliferation [105]. In contrast, suppressing the CXCR4/mTOR signaling pathway in gastric cancer cells induces autophagy-based cell death and inhibits the metastasis [106]. Considering the pro-metastatic role of autophagy, during metastasis, the tumor cells proliferate in the absence of ECM and then circulate within vascular systems to colonize to the secondary tissues [107].

### Exosomes and autophagy in epithelial-to-mesenchymal transition

The tumorigenic effects of tumor-derived exosomes were mainly attributed to the hypothesis that these vesicles are involved in epithelial-to-mesenchymal transition (EMT) process [108–110]. This process causes the loss of cell–cell adhesion, induction of cell polarity, and consequently cell motility and invasiveness [111]. EMT process is characteristic of aggressive tumors. As such, the cells, which have acquired EMT have a tendency to indwell far from the site of origin to a new location. Cho and co-worker showed that exosomes released from ovarian cancer cell lines contribute to induce EMT phenotype in adipose tissue-derived MSCs [109]. Co-culture of MSCs with cancer cell-derived exosomes augmented the expression of alpha-SMA and SDF-1 and TGF-β, suggesting the myofibroblastic phenotype of MSCs via activating the intracellular TGF-β signaling pathway [109]. Garnier et al. have revealed that cancer cells induce mesenchymal phenotypes in cells, which consequently produce exosomes containing tissue factor [112]. In addition, exosomes released during bone marrow-EMT are capable of facilitating multiple myeloma progression in an animal model [113]. This indicates that the EMT-cells produce paracrine factors that influence neighboring cells, consequently inducing resistance in tumor cells.

Autophagy has also been shown to promote EMT phenotype in cells. Autophagy regulation elicits a signaling switch from a mesenchymal phenotype to an epithelial-like form in tumor cells. EMT-activated tumor cells could recruit autophagy machinery in action for their survival under different stress conditions following the metastasis development [114, 115].

### Exosomes and autophagy in tumor angiogenesis

Angiogenesis, the development of new blood networks from the pre-existing vessels, is an essential factor for the growth and metastasis of solid tumors [116]. Hypoxia, which is frequently seen in the tumor environment is a key mediator of angiogenesis [117]. Under the hypoxic condition, autophagy flows through AMPK activity, independent of the HIF-1α pathway that keeps energy balance in cells [118]. In this context, the exosomes biogenesis is induced through activation of the HIF-1α pathway, which promotes angiogenesis [81]. Tumor-derived exosomes modulate endothelial cells to release various growth factors and cytokines, and enable pericytes to undergo migration via PI3K/AKT signaling pathway, which consequently supports angiogenesis [119]. Within the same vein, malignant mesothelioma cells release exosomes that could promote the motility of angiogenic cells and tumor growth via vascular rearrangement and upregulation of angiogenesis [120]. Svensson and colleagues showed that glioma cells release exosome-like vesicles, which could transfer tissue factor and promote angiogenesis by up-regulating protease-activated receptor 2 in epithelial cells [121]. In this regard, under hypoxic conditions, the tumor cells potentially could produce exosomes with pro-angiogenic and pro-metastatic cargo, indicating the compensatory response of tumor cells to the hypoxic stress by stimulating the formation of the vascular bed in the secondary tissue and facilitating the metastasis [122, 123].

In the tumor environment, endothelial cells (ECs) confront with stress conditions including hypoxia, low blood and glucose levels, and nutrient deprivation, which collectively cause the alternations in vascular function and structure [124]. Tumor vessels are different from normal tissues and represent different diameters, more permeability and less stability [125]. These features result in disrupting blood supply and limit availability to oxygen and nutrients.

The key role of autophagy in ECs has been previously well-documented [124], mainly, the ECs recruit autophagy to balance the energy and adapt to stress conditions [126]. A work by Maes and colleague showed that Chloroquine (CQ), an autophagy inhibitor, is capable of promoting tight junctions between ECs, which decreases the invasion and metastasis of tumor cells [127]. Under hypoxic condition, autophagy flux is increased in ECs, which is concurrent with the induction of HIF-1α and VEGF signaling pathways [126]. As mentioned above, increasing autophagy flux in ECs in the tumor microenvironment contributes to maintaining homeostasis. Therefore, the depletion of Atg5 in ECs could intensify the abnormality in the function of tumor vessels indicating the pivotal role of autophagy in ECs homeostasis [127].

The coordination between autophagy and angiogenesis represents some discrepancies. For instance, Rapamycin-induced autophagy promotes angiogenesis in HUVECs through inducing AMPK/Akt/mTOR signaling pathway [128]. However, in ischemic myocardium model of acute myocardial infarction (AMI) in mice, ROS-ER stress/autophagy axis promotes angiogenesis in cooperation with vascular endothelial growth factor A (VEGF-A) in endothelial cells [129]. More recently, the anti-angiogenic effect of autophagy in ECs has been reported when ECs treated with mebendazole. This could be a new target for cancer therapy [130]. Collectively, autophagy is cytoprotective and essential to redox homeostasis, which mediates the adaptive function of ECs to blood flow and energy depletion. Despite the deep focus on understanding key signaling mechanisms, the detailed relationship among exosome, autophagy, and angiogenesis pathways are still not clear.

### Exosomes and autophagy in tumor suppression

Several studies demonstrate that exosomes derived from tumor cells play pivotal roles in promoting tumorigenesis [131]. Nevertheless, based on the type of exosome source, this phenomenon may vary. For example, Wu et al. reported that exosomes from umbilical cord Wharton’s jelly MSCs inhibit proliferation of bladder tumor cells through decreasing phosphorylation of Akt protein kinase and promoting caspase-3 [132]. Furthermore, exosomes derived from adipose MSCs have shown to suppress prostate cancer through the distribution of miR-145 and by inhibition of the activity of Bcl-xL protein and stimulating apoptosis via the caspase-3/7 pathway [133]. Similarly, human bone marrow MSCs release exosomes that were reported to suppress proliferation and induce apoptosis in ovarian tumor cell lines, liver cancer and Kaposi’s sarcoma [134].

As mentioned above, autophagy eliminates the intracellular harmful proteins and organelles for keeping cells normal and to inhibit tumorigenesis [135]. However, it was demonstrated that autophagy represents a dual role also in tumor cell dynamic, contributing as pro-metastatic and anti-metastatic modulator [78]. In the early stage of metastasis, autophagy shields the tumor cells against necrosis and inflammation, and decreases invasion and motility of tumor cells, thus suppresses the tumor growth. Conversely, in progressive metastasis stage, autophagy shows a pro-metastatic property, therefore, promotes cell survival and migration toward secondary tissues [78]. As a result, inhibition of autophagic genes including LC3 and Beclin-1 inhibits proliferation, movement, invasion, and increases apoptosis rate of breast cancer cells [96]. Dysfunction in autophagic elements such as ATG12, ATG9B, ATG4 and ATG5 may result in tumor initiation [136]. Furthermore, autophagy induced by nutrient starvation and mTOR inhibition has been shown to suppress cell migration in glioblastoma cells in vitro. However, the inhibition of ATG5, ATG7, and Beclin 1 augmented the migration and invasion in glioblastoma cells [137], indicating that autophagy inhibition may serve as potential anti-tumor therapy approach.

### Therapeutic resistance related to exosomes and autophagy

Besides a normal status, stressors such as radiotherapy are capable of affecting the dynamic of exosomal [131], and autophagic pathway in cancer cells [138, 139]. For example, we have recently found that radiotherapy promotes biogenesis and secretion of exosomes in breast cancer cells in vitro [140]; which may contribute to therapeutic resistance [131]. Additionally, radiotherapy causes alterations in the exosome cargo of radiated cells. These exosomes, when co-cultured with non-radiated cells, could induce tumorigenesis in the recipient cells and promotes cancer resistance [141, 142]. Radiotherapy may induce autophagy-based cell death in normal and cancer cells [143]. In this regard, Daido and co-workers found that irradiation of glioblastoma cells caused cell death via autophagy independent of apoptosis [144]. The underlying mechanisms that associate radiotherapy and autophagy-related death have not been well known, however, the mTOR pathway and the endoplasmic reticulum stress could have a role in irradiation/autophagy-related cell death [145]. The irradiated cells recruit autophagy in action to promote resistance against radiotherapy. Subsequently, the inhibition of autophagy causes radio-sensitization through the elimination of injured molecules in the tumor cells, which may serve as a mean to increase the efficacy of radiotherapy [146, 147]. In the case of chemotherapy, a growing body of literature has confirmed that the chemotherapy activates exosome biogenesis pathway and alters exosomes cargo, thus, induces exosome-based chemoresistance [148–150]. Increased exosome secretion may provide a way to cells for escaping from the cytotoxic effects of drugs and to promote tumor progression [148]. Different mechanisms have been reported for exosome-mediated chemoresistance. For example, non-coding RNAs such as miRNAs and lncRNAs cargo of exosomes secreted from cancer cells contribute to cellular chemoresistance against Paclitaxel [151], and Tamoxifen [152]. According to literature various signaling pathways can be activated against exosomal RNAs to initiate resistance against drugs. This may involve TGF-β, Wnt, receptor tyrosine kinase (AXL), c-MET, anti-apoptosis, and cell cycling pathways in different tumor cell lines [153, 154].

Similarly, autophagy is also induced against chemotherapy treatment to cells and safeguards cells from the cytotoxic effects of chemical drugs and promotes chemoresistance [139, 155]. In support, Garbar et al. showed that chemotherapy drugs can induce and increase the autophagy in tumor cells [156]. However, a growing body of evidence showed the anticancer role of autophagy against chemotherapy, where chemotherapy drugs caused autophagy-mediated death in tumor cells [157, 158]. Such paradoxical roles could be due to the heterogeneous composition of drugs and type of cell lines used in different studies [155].

Cooperation between exosomal and autophagy has been studied in chemotherapy treatment experiments. For example, Yin and colleagues found that treatment of ovarian cancer cell lines with cisplatin increases the secretion of exosomes containing annexin A3 in cell culture supernatants and also in sera from ovarian cancer patients [159]. Annexin A3 expression is associated with the autophagy flux in tumor cells [160]. Rotenone, a chemical pesticide was shown to induce stress in breast and prostate cancer cell lines, which caused the induction of autophagy and exosome secretion [68]. In another work, treatment of gefitinib to EGFR-mutant PC-9 cells caused the elevated exosome secretion which promotes autophagy flux in recipient cancer cells [161]. In the case of cancer therapy, understanding how and when cancer cells engage these pathways to survive tumors is fundamental to improved cancer management.

### Exosomal and autophagic proteins as potential cancer biomarkers

As discussed above, the intracellular vesicles, autophagic compartments, and exocytosis play essential roles in cancer progression. Over-activated in tumor cells, key protein components of the exosomal and autophagy pathways could be used as a biomarker for various cancers [161, 162]. In this section, we focus on the biomarker application of exosomal and autophagy-related protein in cancer detection.

As mentioned, the exosomal system has been found to facilitate tumor cell proliferation, metastasis, invasion, and angiogenesis by transferring biomolecules such as various nucleic acids, proteins, and lipids [163]. Exosomes can be distributed through bio-fluids, such as plasma, urine, bile, breast milk, cerebrospinal fluid, amniotic fluid, and saliva. Therefore, capturing cancer-specific exosomes from body fluids may serve as a valuable source for obtaining information about the tumor environment/status [164]. This could serve as a non-invasive source of biomarkers and may aid an alternative source to liquid-biopsy for cancer diagnosis.

The exosomal cargo have been identified in different organisms and are presented in various online databases including Vesiclepedia (www.microvesicles.org) and ExoCarta (www.exocarta.org), and Evpedia (http://evpedia.info) [165, 166]. For example, ExoCarta database has presented about 5000 mRNA records, 41,860 protein records, and 1116 lipid records in exosomes from 286 different studies according to the latest release in 2019. These data further provide us with information about exosome biogenesis, extracellular trafficking, uptake, clinical application, and biological functions in target cells [167]. In this regard, besides pre-clinical biomarker investigations (Table 1), there is increasing interest in clinical trials to evaluate biomarker application of exosomes in cancer prognosis and diagnosis that presented in Table 2.

**Table 1:** Exosomal and autophagic proteins as potential biomarkers

Cancer type	Exosomal proteins as biomarker	Autophagic proteins as biomarker
Bladder	α6-integrin, Basigin, TACSTD2, Mucin4, EDIL-3, EPS8L2, MUC-1 [169]	NR
Breast	Survivin, Survivin-2B, CEA, Tumor antigen15-3 [170]	LC3β [171], LC3α [172], ULK-1 [173], Beclin-1 [174], FIP200 [175]
Cervical	ATF1, RAS [176]	NR
Colorectal	CEA [110]	Beclin1, LC3β [174]
Gastric	NR	ULK1, Beclin 1, ATG3, ATG10 [161]
GBM	EGFRvIII [177]	NR
Melanoma	CD63, Caveolin1, TYRP2, VLA-4, HSP70 [110]	LC3β [178]
lung	EpCAM, EGFR, CEA, LRG-1 [179]	LC3β [180], Beclin-1 [174]
Ovarian	MAGE3/6, Claudin-4, L1CAM, TGFβ1, CD24, ADAM10, EMMPRIN [181]	NR
Pancreatic	GPC1, MIF [182]	NR
Prostate	Survivin, PTEN, Transmembranes, Protease, ITGB1, Serine2-ETS, β-catenin, PSA, PCA3, PSMA, ITGA3 [183]	NR

NR means not recorded

**Table 2:** Cancer-related clinical trials for exosomal and autophagic biomarkers

Cancer	Exosomal biomarker		Autophagic biomarker	
	Status	Identifier	Status	Identifier for
Advanced Cancers	NR	NR	Active, not recruiting Active, not recruiting	NCT02042989 NCT01266057
Bone Metastases	Recruiting	NCT03895216	NR	NR
Breast	Not yet recruiting Withdrawn	NCT03974204 NCT01344109	Unknown Terminated Recruiting	NCT01292408 NCT00765765 NCT03774472
Bladder	NR	NR	Not yet recruiting	NCT03254888
Cholangiocarcinom	Recruiting	NCT03102268	NR	NR
Gallbladder	Recruiting	NCT03581435	NR	NR
Gastric	Unknown	NCT01779583	NR	NR
Kidny	NR	NR	Terminated	NCT01144169
Liver			Recruiting	NCT03037437
Lung	Recruiting Recruiting Unknown	NCT03830619 NCT03228277 NCT02869685	Completed Completed Completed	NCT00969306 NCT00728845 NCT01649947
Malignant Solid Tumor	NR	NR	Active, not recruiting	NCT01023737
Melanoma	NR	NR	Recruiting Terminated	NCT03754179 NCT00786682
Ovarian	Recruiting	NCT03738319	NR	NR
Pancreatic	Completed	NCT03032913	NR	NR
Prostate	NR	NR	Terminated Terminated Active, not recruiting Active, not recruiting	NCT02421575 NCT00786682 NCT02339168 NCT01480154
Colorectal	Recruiting	NCT03874559	Completed Completed Active, not recruiting	NCT01006369 NCT01206530 NCT02316340
Thyroid	Recruiting	NCT03488134	NR	NR
Thyroid	Active, not recruiting	NCT02862470	NR	NR

NR means not recorded

A survey on the clinical trial database (ClinicalTrials.gov) showed 14 recorded studies about analysis of exosomes as cancer biomarker up to February 2020. In addition, due to the pivotal roles of autophagy in cancer biology, recent studies have suggested the biomarker potential of autophagic proteins in cancer prognosis and diagnosis (Table 1). Of note, common autophagy markers including Beline-1, p62, and LC3β used in a variety of experiments to assess autophagy flux in vitro and in vivo [168], may provide useful details for the cancer detection. Following the para-clinical researches, clinical trials have aimed to study autophagy in cancer, however, when autophagy is used as a keyword in clinical trials site (ClinicalTrials.gov), 27 records are prepared (Table 2) up to February 2020. Although these studies have proposed the clinical application of both exosomal and autophagic proteins in cancer management, future studies are required to validate the predictive potential of biomarkers.

## Conclusion

Exosomal and autophagy pathways support cells to response against stress conditions and communicate to neighboring cells. Both processes play pivotal roles in homeostasis and metastasis of tumor cells. Exosome biogenesis pathway is linked to autophagy in different ways including the fusion of autophagic vesicles with the lysosome to degrade cargo where autophagy-related proteins may also contribute to exosome generation and secretion. Coordination between exosomal and autophagy pathways regulates tumor cells responses against stress conditions. Due to involvement in tumor biology, the biomarker application of the regulatory proteins of these pathways has been suggested. However, detailed mechanisms underlying the crosstalk between exosome biogenesis and autophagy remain still unclear. In this regard, there exist some questions that should be answered in further experiments: Which mechanisms are involved in directing MVBs to the lysosome, autophagosome, and the plasma membrane? How the fate of autophagosomes is differentially regulated? Are autophagosomes cargo sorted into exosomes in amphisomes?

Understanding the crosstalk between endomembrane organelles and vesicular trafficking and the molecular mechanisms involved, may expand our insight into cooperative functions of autophagy and exosomal pathways, thus targeting these pathways may help to develop effective therapies against lysosomal diseases including cancers and beyond.

## Data Availability

Not applicable.

## Abbreviations

ALIX: ALG-2-Interacting Protein X
ANXA2: Annexin A2
CMA: Chaperone-Mediated Autophagy
CML: Chronic Myeloid Leukemia
CQ: Chloroquine
ECs: Endothelial Cells
ECM: Extracellular Matrix
EMT: Epithelial-to-Mesenchymal Transition
ER: Endoplasmic Reticulum
ESCRT: Endosomal Sorting Complexes Required for Transport
EVs: Extracellular Vesicles
GAIP: G Alpha Interacting Protein
GIPC: GAIP-Interacting Protein C-terminus
IL-1β: Interleukin 1β
ILVs: Intraluminal Vesicles
IVS: Intracellular Vesicular System
LAP: LC3β-Associated Phagocytosis
MHC: Major Histocompatibility Complex
MMPs: Matrix Metalloproteinase
MSCs: Mesenchymal Stem Cells
mTOR: Mechanistic Targeting of Rapamycin
mTORC1: mTOR Complex 1
MVB: Multivesicular Body
NR: not recorded
NSF: NEM-Sensitive Factor
PA: Phosphatidic Acid
PE: Phosphatidyl Ethanolamine
PIKfyve: finger-containing Phosphoinositide Kinase
PLP: Proteolipid Proteins
ROS: Oxygen Species
SNAREs: NSF Attachment Protein Receptor
TSG101: Tumor Susceptibility Gene 101
ULK1: Unc-51-like Kinase 1
VEGF-A: Vascular Endothelial Growth Factor A
